# Evaluation of the clinical efficacy of 1.2% atorvastatin in the treatment of periodontal intraosseous defects by CBCT: A randomized controlled clinical trial

**DOI:** 10.15171/joddd.2019.029

**Published:** 2019-10-07

**Authors:** Prerna Y. Shirke, Abhay P. Kolte, Rajashri A. Kolte, Pranjali V. Bawanakar

**Affiliations:** ^1^Department of Periodontics & Implantology, VSPM Dental College and Research Centre, Nagpur, India

**Keywords:** CBCT, intrabony defects, periodontal regeneration, periodontitis, scaling and root planing

## Abstract

***Background.*** Atorvastatin (ATV), which belongs to the statin class of drugs, is the formidable inhibitor of 3-hydroxy-2- methyl-glutaryl coenzyme A reductase. This clinical trial evaluated and compared the clinical and radiographic changes in chronic periodontitis (CP) patients, obtained through 1.2% ATV as an adjunct to scaling and root planing (SRP) in the treatment of intraosseous defects.

***Methods.*** Twenty CP patients, with a minimum of one pair of bilateral intraosseous, were randomly selected for this splitmouth study. Group 1 included 20 sites treated with SRP and subgingival delivery of a placebo gel, whereas an equal number of sites in group 2 were treated by SRP along with subgingival delivery of 1.2% ATV gel. The plaque index (PI), modified sulcus bleeding index (mSBI), probing pocket depth (PPD) and clinical attachment level (CAL) were evaluated at baseline and 3- and 6-month intervals, while the intraosseous defect was assessed at baseline and 6-month interval using cone-beam computed tomography (CBCT). Paired t-test was used to determine statistical significance.

***Results.*** A greater reduction in the mean PPD and gain in CAL was found in group 2 compared to group 1 at 3- and 6-month intervals. Furthermore, a significantly greater bone fill was obtained in group 2 (1.70±0.54 mm) compared to group 1 (0.22±0.43 mm) after six months.

***Conclusion.*** ATV, as an adjunct to SRP, enhanced periodontal regeneration, as a noninvasive way to treat periodontal intraosseous defects.

## Introduction

Chronic periodontitis (CP) is an inflammatory disease of microbial origin, primarily affecting the supporting tissues of the teeth and resulting in progressive destruction of the periodontal ligament and alveolar bone. Progression of CP is featured by increased osteoclast activity, which leads to the formation of intraosseous defects, and if left untreated, it eventually leads to tooth loss.^1^ Scaling and root planing (SRP) disrupts and eliminates the microbial bioﬁlm from the root surfaces of periodontally diseased teeth. It also removes diseased and necrotic cementum, thus providing a healthy root surface for the healing attachment. Furthermore, it decreases gingival inflammation and periodontal pocket depth, leading to a gain in the clinical attachment level.^2^ Nevertheless, to amplify the outcomes of SRP, an agent is needed, which can act as an adjuvant and also help effectively in gaining clinical attachment level, inhibit resorption of the alveolar bone and stimulate new bone formation.^3^

Statins (3-hydroxy-2-methyl-glutaryl coenzyme A reductase inhibitors) are a group of lipid-lowering drugs that are widely used to prevent cardiovascular events. Nowadays, the use of statins is gaining momentum in the management of periodontal diseases owing to their anti-inflammatory and immunomodulatory effects. Statins have various additional benefits, including the formation of bone morphogenic protein (BMP)-2, promotion of osteogenesis by inhibiting osteoblast apoptosis and suppression of osteoclastogenesis. Statins directly affect osteoclasts, which predominantly depend upon the inhibition of the formation of intermediates that are required to prenylate proteins that inhibit the osteoclastic activity. Statins act through osteoblast-osteoclast cross-talks and involve the RANKL (Receptor activator of nuclear factor kappa-Β ligand)/OPG (osteoprotegerin) system. Statins have been found to down-regulate the production of many proinflammatory cytokines, including interleukin (IL)-1α-induced IL-6 and IL-8 in the epithelial cells in a dose-dependent manner. Moreover, they also decrease the secretion of matrix metalloproteinases (MMPs), such as MMP-1, MMP-2, MMP-3 and MMP-9 in vitro. Thus, statins might reduce the fierce immune response and thereby alveolar bone destruction.^4^ Atorvastatin (ATV) enhances osteoblastic production of OPG, a crucial osteoblast-derived cytokine that neutralizes RANKL and prevents the formation and activation of osteoclasts by promoting osteoblastic differentiation. ATV therapy decreases tumor necrosis factor (TNF)-α production in lipopolysaccharide-activated monocytes, substantiating the anti-inﬂammatory properties of this class of drugs.^5^ Despite all these beneficial effects of the ATV in the treatment of CP, there appears to be paucity of literature about the use of ATV in periodontal regeneration. The preliminary results seem to be encouraging in terms of regeneration; therefore, it was felt necessary to further study this statin in the treatment of periodontal intraosseous defects.

CBCT is one of the most advanced and accurate methods for the evaluation and assessment of periodontal regeneration; however, very few studies have used CBCT to assess regeneration.^6-8^ Moreover, in these studies, periodontal regeneration was achieved surgically, using either bone grafts, guided tissue regeneration membranes or platelet-rich fibrin (PRF). The need to achieve and assess regeneration non-invasively and cost-effectively prompted us to design the current randomized clinical trial to evaluate and compare the efficacy of 1.2% ATV gel as an adjunct to SRP and SRP with a placebo gel in the treatment of periodontal intraosseous defects clinically and radiographically, using CBCT.

## Methods

### Study Design

This split-mouth randomized controlled clinical trial was conducted from October 2017 to November 2018 at the outpatient Department of Periodontology of our institute in accordance with the Helsinki Declaration of 1975, as revised in 2013. The study was also approved by the Institutional Ethics Committee. This clinical trial was registered at Clinical Trial Registry–India, being the primary register of the WHO International Clinical Trials Registry Platform. The registration number allotted for this trial was CTRI/2017/10/010289. A written informed consent form was obtained from all the participating patients.

### Sample Size 

The sample size was calculated based on the results of a study by Zamet et al.^11^ Power analysis resulted in an effect size of 0.7 for the number of patients and defects. With 90% power and 95% confidence level, a sample size of 20 patients, with at least one pair of bilateral intraosseous defects, was essential to achieve a significant effect.

**Figure 1:**
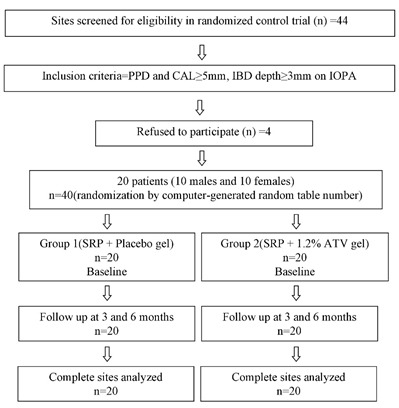
Consolidated Standards of Reporting Trials (CONSORT) flowchart.

Hence, 20 CP patients (10 males and 10 females) with a mean age of 35.7 years (range: 30‒45 years) were enrolled in the study as classified on the basis of the 1999 consensus classification of periodontal disease^9^ (Figure 1).^10^ A total of 20 pairs of intraosseous defects, including 12 pairs of mandibular defects and 8 pairs of maxillary defects were selected preferentially in molars for maintaining the uniformity in defect morphology.

The inclusion criteria consisted of probing pocket depth (PPD) and clinical attachment level (CAL)≥5 mm, along with the presence of at least one pair of similar intraosseous defect in a systemically healthy patient, which was ≥3 mm deep as identified on a diagnostic intraoral periapical radiograph (IOPA). Patients with suspected or known allergy to the ATV/statin group, those on systemic ATV/statin therapy, with a history of periodontal treatment in previous 6 months, aggressive periodontitis patients, tobacco chewers, smokers, alcoholics, diabetic patients and pregnant or lactating women were excluded from the study.

### Intra-examiner Calibration

The clinical parameters for five pairs of intraosseous defects were evaluated by a single examiner (AK), while the radiographic parameters for five pairs of intraosseous defects were assessed by another single examiner (RK) on CBCT images. Both the clinical and radiographic measurements were performed again after 48 hours. With the use of Kappa value and significance test for intra-examiner calibration in the measurements of clinical and radiographic parameters, calibration was established when ≥90% of the recordings could be reproduced within a 1-mm difference.

### Clinical and Radiographic Measurements

Plaque index (PI),^12^ modified sulcus bleeding index (mSBI),^13^ PPD and CAL were obtained at baseline and 3- and 6-month intervals. A UNC-15 (University of North Carolina 15, Hu-Friedy®, Chicago, IL, USA) periodontal probe was used for PPD and CAL measurements, which were rounded off to the nearest millimeter mark. CBCT (Orthophos® XG 3D/Ceph, Sirona Dental Systems GmbH, Germany) was used to measure the intraosseous defect sites at baseline and after 6 months. This incorporated the measurement of the bone defect height [CEJ–BD (base of the defect)], the level of the alveolar crest [CEJ–AC (alveolar crest)], the bone defect depth (AC‒BD) and the mesiodistal (MD) and buccolingual (BL) bone defect width. A line perpendicular was drawn from the AC to the root surface, and the intersection point across the root surface was considered as AC. The distance from the point AC to the base of the defect (AC‒BD) was considered as the intraosseous defect depth. The distance from the point AC to the alveolar crest (AC) was considered as the MD width of the intraosseous defect. The BL width was measured in the axial plane as the horizontal distance between the most coronal point for the buccal and lingual alveolar crest. A slice thickness of 0.2 mm was used for CBCT analysis.

### Preparation of 1.2% ATV Gel

ATV gel was prepared according to the method described in a previous study.^14^ Methylcellulose gel was prepared by adding the required amount of distilled water to an accurately weighed amount of methylcellulose. The vial was heated to 50‒60ºC and kept in a mechanical shaker to obtain a clear solution. A weighed amount of ATV was then added to the obtained solution and dissolved completely to form a uniform phase of methylcellulose, solvent and drug. The placebo gel contained only methylcellulose gel without adding ATV.

### Clinical Procedure

The patients were randomly assigned to two groups. The randomization process was made by the statistical unit by a computer-generated random table number, and the investigators were not involved in the randomization process and were unaware of the assigned group in all the outcome evaluations. The clinical procedure consisted of SRP until the root surface was considered by the operator (PS) to be clean and free of any deposits. The patients in group 1 were treated by SRP, followed by the subgingival placement of 0.1 mL of placebo gel, while the patients in group 2 were treated by SRP, followed by the placement of 0.1 mL of 1.2% ATV gel subgingivally. The patients were instructed not to chew hard or eat sticky food or brush near the treated areas for one week. The patients were followed up to 3 and 6 months, and supragingival deposits, if present, were removed.

### Statistical Analysis

The analysis of the data was carried out using the SPSS 20. The P-value<0.05 was considered statistically significant. All the clinical parameters were presented as mean ± SD. PI and mSBI were compared at different time intervals, using the Friedman test and ANOVA. The changes in the index parameters between the two groups were compared with Wilcoxon’s rank sum test. The PPD and CAL were compared at different time intervals with one-way ANOVA. The change in these clinical parameters at 3 and 6 months from the baseline were compared between the two groups by paired t-test. The CEJ‒BD, CEJ‒AC, AC‒BD, MD and BL distances on CBCT images were compared between the baseline and 6 months using the paired t-test. The mean change at 6 months from the baseline was compared between the two groups with paired t-test.

## Results

### Clinical Parameters

**Figure 2:**
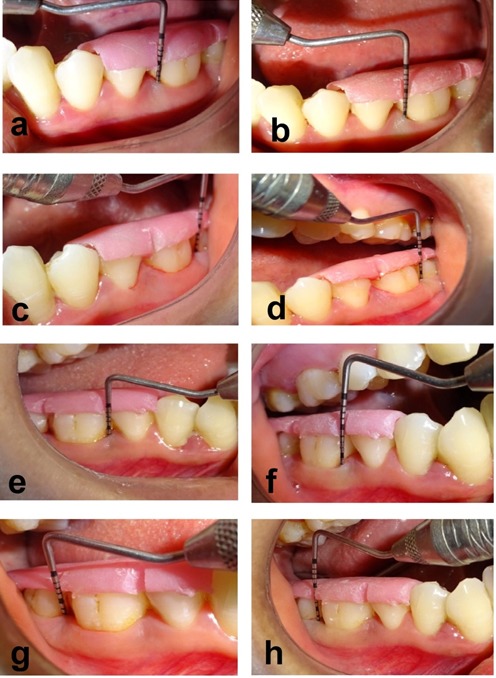
Comparison of PPD at baseline and after 6 months between groups 1 and 2. (a) PPD in group 1 at baseline. (b) PPD in group 1 at 6 months. (c) PPD in group 1 at baseline. (d) PPD in group 1 at 6 months. (e) PPD in group 2 at baseline. (f) PPD in group 2 at 6 months. (g) PPD in group 2 at baseline. (h) PPD in group 2 at 6 months.

In the present split-mouth study, there was a highly significant reduction in the PI and mSBI at 3 and 6 months. Baseline full-mouth PI was 1.76±0.22, while at 3 months, it decreased to 1.04±0.23, and at 6 months, the mean PI score was 0.61±0.18. The mean mSBI score decreased from 2.78±0.66 at baseline to 1.39±0.19 at 3 months and 1.11±0.08 at 6 months. At 6 months, the mean PPD reduction was 1.80±0.41 mm in group 1 and 3.05±0.61 mm in group 2. There was a statistically significant reduction in the PPD for both groups at 6 months when compared to baseline. The mean CAL gain at 6 months in group 1 was 1.90±0.55 mm, with 3.35±0.74 mm in group 2. There was a statistically significant CAL gain in both groups at 6 months when compared to the baseline. There was a statistically significant CAL gain at 3 and 6 months in group 2 when compared to group 1 (P<0.0001) (Tables 2 and 3) (Figure 2).

**Table 1:** Intra-group comparison of the measurements of clinical parameters (mean ± SD) at baseline and 3 and 6 months later (in mm)

**Parameters**	**Group 1**		**Group 2**	
	**Mean ± SD**	P-value*	**Mean ± SD**	P-value*
**PPD (in mm)**				
**Baseline**	7.45 ± 0.51		7.50 ± 0.51	
**3 months**	6.20 ± 0.69	<0.0001 (HS)	5.60 ± 0.59	<0.0001 (HS)
**Baseline**	7.45 ± 0.51		7.50 ± 0.51	
**6 months**	5.65 ± 0.58	<0.0001 (HS)	4.45 ± 0.51	<0.0001 (HS)
**CAL (in mm)**				
**Baseline**	7.80 ± 0.52		7.90 ± 0.55	
**3 months**	6.50 ± 0.76	<0.0001 (HS)	5.80 ± 0.62	<0.0001 (HS)
**Baseline**	7.80 ± 0.52		7.90 ± 0.55	
**6 months**	5.9 0± 0.64	<0.0001 (HS)	4.55 ± 0.51	<0.0001 (HS)

*Obtained using paired t-test; HS: Highly Significant

**Table 2:** Intra-group comparison of the measurements of radiographic parameters (mean ± SD) at baseline and 6 months later (in mm)

**Parameters**	**Group 1**		**Group 2**	
	**Mean ± SD**	P-value*	**Mean ± SD**	P-value*
**CEJ-BD (in mm)**				
**Baseline**	10.16 ± 0.59		10.35 ± 0.63	
**6 months**	9.96 ± 0.64	<0.0001 (HS)	8.20 ± 0.61	<0.0001 (HS)
**CEJ-AC (in mm)**				
**Baseline**	5.33 ± 0.68		5.49 ± 0.78	
**6 months**	5.34 ± 0.58	0.940 (NS)	5.04 ± 0.58	0.012 (S)
**AC-BD (in mm)**				
**Baseline**	4.84 ± 0.37		4.87 ± 0.45	
**6 months**	4.62 ± 0.37	0.337 (NS)	3.16 ± 0.29	<0.0001 (HS)
**MD (in mm)**				
**Baseline**	2.03 ± 0.51		2.63 ± 0.65	
**6 months**	1.81 ± 0.53	0.053 (NS)	1.75 ± 0.59	<0.0001 (HS)
**BL (in mm)**				
**Baseline**	5.20 ± 1.31		4.89 ± 1.63	
**6 months**	4.35 ± 1.43	0.003 (S)	3.53 ± 1.66	<0.0001 (HS)

*Obtained using paired t-test; NS: Not Significant, S: Significant, HS: Highly Significant

**Table 3:** Inter-group comparison of the measurements of clinical and radiographic parameters (mean ± SD) at 6 months (in mm)

	**Parameters**	**Group 1**	**Group 2**	P-value*
		**Mean ± SD**	**Mean ± SD**	
**Clinical** **Parameters**	**Mean PPD reduction** **(in mm)**	1.80 ± 0.41	3.05 ± 0.61	<0.0001(HS)
	**Mean CAL gain** **(in mm)**	1.90 ± 0.55	3.35 ± 0.74	<0.0001(HS)
**Radiographic** **Parameters**	**Mean change in CEJ-BD** **(in mm)**	0.20 ± 0.69	2.15 ± 0.49	<0.0001 (HS)
	**Mean change in CEJ-AC** **(in mm)**	0.02 ± 0.89	0.44 ± 0.72	0.120 (NS)
	**Mean change in AC-BD** **(in mm)**	0.22 ± 0.43	1.70 ± 0.54	<0.0001 (HS)
	**Mean change in MD** **(in mm)**	0.29 ± 0.63	0.68 ± 0.48	0.085 (NS)
	**Mean change in BL** **(in mm)**	1.13 ± 0.85	1.36 ± 0.47	0.073 (NS)

*Obtained using paired t-test; NS: Not Significant, HS: Highly Significant

### Radiographic Parameters of Intraosseous Defects on CBCT

### Intraosseous Defect Depth Height (CEJ‒BD) 

**Figure 3:**
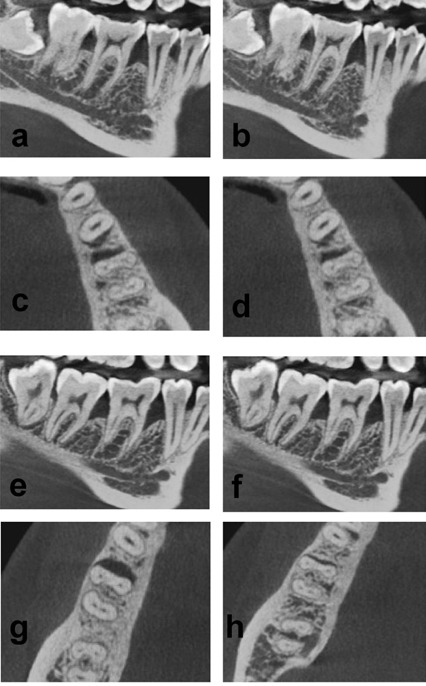
Comparison of CBCT images at baseline and after 6 months between groups 1 and 2. (a) Intraosseous defect in the sagittal view in group 1 at baseline. (b) Reduction in the intraosseous defect in the sagittal view in group 1 at 6 months. (c) The intraosseous defect in the transverse view in group 1 at baseline. (d) Reduction in the intraosseous defect in the transverse view in group 1 at 6 months. (e) The intraosseous defect in the sagittal view in group 2 at baseline. (f) Reduction in the intraosseous defect in the sagittal view in group 2 at 6 months. (g) The intraosseous defect in the transverse view in group 2 at baseline. (h) Reduction in the intraosseous defect in the transverse view in group 2 at 6 months.

At 6 months, there was a decrease in the mean CEJ‒BD, indicating a bone fill of 0.20±0.69 mm and 2.15±0.49 mm in groups 1 and 2, respectively. Significantly higher bone fill was observed in group 2 as compared to group 1 (Tables 2 and 3) (Figure 3).

### Level of the Alveolar Crest (CEJ‒AC) 

The difference in the values of CEJ‒AC from the baseline to 6 months indicates the change in the level of the alveolar crest. At 6 months, the mean values significantly increased by 0.02±0.89 mm in group 1, and a statistically significant decrease of 0.44±0.72 mm was observed in group 2. This indicates the resorption of the alveolar crest in group 1 and an increase in the height of the crest in group 2 (Tables 2 and 3) (Figure 3).

### Depth of the Intraosseous Defect (AC-BD) 

Defect depth reductions of 0.22±0.43 and 1.70±0.54 mm were observed in groups 1 and 2, respectively. This shows significantly higher defect depth reduction in group 2 as compared to group 1 (Tables 2 and 3) (Figure 3).

### Mesiodistal Width (MD) 

At 6 months, the MD width reduction was higher in group 2 as compared to group 1, which was not statistically significant (Tables 2 and 3) (Figure 3).

### Buccolingual Width (BL) 

When the BL width reduction at 6 months was compared between the two groups, it was higher in group 2 as compared to group 1, which was not statistically significant (Tables 2 and 3) (Figure 3).

## Discussion

The potential role of statins in periodontal regenerative therapy has been established over time. Several studies have examined periodontitis and statins in animals, with the results showing beneficial effects on

the periodontium.^15-18^ There have been very few studies reporting the use of 1.2% ATV as LDD in the intraosseous defects in patients with CP.^14,19-21^ The present clinical trial showed signiﬁcant improvements in the clinical parameters and bone fill on CBCT when 1.2% ATV gel was used as an adjunct to SRP for the treatment of intraosseous defects in CP compared to a placebo gel. Systemic administration of 10 mg of ATV reduced oxidative stresses more efficiently than 40 mg of simvastatin (SMV) in patients with type II diabetes mellitus, suggesting that ATV is more antioxidant than SMV.^22^ To the best of the author’s knowledge, this is the first clinical trial which evaluated bone fill, using CBCT after treating intraosseous defects with 1.2% ATV gel in combination with SRP in CP. The current study considered the technique of subgingivally delivering 0.1 mL of ATV gel per site directly into the intraosseous defects in individuals with CP as previously demonstrated.^14^ Local delivery might offer important benefits over the systemic regimen in terms of adverse reactions and patient compliance with the reduced dosage, fewer applications, and high patient acceptability, with the advantages of high concentrations at the required site. Statins prevent not only periodontal tissue breakdown in animal models^16^ but also have beneficial effects on the alveolar bone recovery after ligature-induced alveolar bone resorption in rats.^17^ A study suggested that patients with CP under statin medication had 37% lower periodontal pockets than those without statin medication.^23^ One of the most important clinical outcome variables in regenerative studies is PPD and CAL changes following regenerative therapy. In our study, group 2 exhibited a greater reduction in PPD as compared to group 1, which can be attributed to the fact that statins inhibit inflammatory cells and MMP levels,^24^ highly correlating to PPD and bleeding on probing^25^ and playing a key role in the connective tissue destruction in periodontal disease. Similar improvements in PPD and CAL were obtained in previous studies.^14,19^ In our study, reduction in CEJ‒BD was higher in group 2 as compared to group 1, which signifies a gain in the level of alveolar bone, indicating the important role of ATV in periodontal regeneration. The change in the CEJ‒AC distance denotes a change in the level of the alveolar crest. In our study, the CEJ‒AC distance increased in group 1 but decreased in group 2 at the end of 6 months. This indicates that the alveolar crest resorption was higher in group 1, which was deprived of the delivery of 1.2% ATV gel. This finding was consistent with the previous results where the authors found that systemic ATV administration for 3 months in patients with periodontal disease resulted in a decrease in the CEJ‒AC distance and tooth mobility, signifying bone gain at the end of the term.^26^ On the contrary, our study showed similar results by local drug delivery, which indicates increased concentration of the drug achieved at the desired site at lower doses compared to the systemic administration. In our study, the defect depth reduction was higher in group 2 than in group 1. ATV is the lipophilic statin that appears to have a more potent bone-sparing effect than hydrophilic statins. This bone fill might be attributed to increased BMP-2 expression during bone regeneration, anti-inflammatory effects and angiogenesis during wound healing.^27^ Improvements in group 1 could be explained by the SRP and oral hygiene instructions provided at baseline. A similar intraosseous defect depth reduction was found using ATV in previous studies.^14,19-21^ In these studies, the defect fill was evaluated on digital radiographs by software using an image analyzer. On the contrary, our study used CBCT, which offers better visualization of the bone defect and has higher accuracy than any other radiographic image modality. CBCT could provide relatively accurate measurements of MD width of the defect and the BL width of the defect, which periapical radiograph cannot show.^28^ The CBCT allowed for an analysis of the buccal and lingual/palatal surfaces and an improved visualization of the morphology of the defect. The CBCT technique allows better visualization of the defect and helps in better preoperative decision making for treatment.^29^ More MD and BL fill was reported in the test group, as compared to the control group, when regeneration of periodontal intraosseous defects was evaluated by a re-entry procedure 9‒13 months after the surgical procedure.^30^ In our study, the regeneration therapy of intraosseous defects was carried out non-surgically using LDD of 1.2% ATV gel in the defects and evaluation of the regeneration was carried out after 6 months by the CBCT and, a surgical re-entry procedure was not performed. This affirms the previous findings that the CBCT technique might obviate surgical re-entry as a technique for assessing the regenerative therapy outcomes.^31^ A retrospective study stated that any statin use for 3 years was not associated with tooth loss rate in the year subsequent to the 3-year period.^32^ Although the pharmacological effects of statins could be accountable for this result, the observed association between the statin use and decreased tooth loss could reflect confounding by unmeasured factors. Lack of control for some potential confounders, such as smoking, and evaluation of different patterns of statin use might have hampered the interpretation of the results. ATV was able to prevent the alveolar bone loss seen in a ligature-induced periodontitis model in Wistar rats.^4^ Hence, both of the above studies were unable to establish a strong link between the use of statins and periodontitis. Our study results confirm the findings of both studies. Comparative evaluation of 1.2% SMV gel and 1.2% ATV gel in the treatment of periodontal intraosseous defects showed that ATV gel resulted in greater improvements in the clinical parameters with a higher percentage of radiographic defect depth reduction as compared to SMV gel.^20^ This can be attributed to the fact that ATV has superior kinetics than SMV. A lower dose of ATV (5 mg), as compared to 10 mg SMV and 40 mg lovastatin, resulted in a 30% reduction in the LDL cholesterol, indicating a strong pharmacokinetic profile and the ability to achieve target therapeutic concentrations.^33^ In a crossover study, it was observed that ATV was more beneficial than SMV in terms of vitamin D concentrations as well as markers of oxidative stress and inflammation in patients with type II diabetes mellitus.^34^ Thus, better pharmacokinetics and potent antioxidant and anti-inflammatory properties can be considered as one of the reasons for superior results in the ATV group as compared to the SMV group.

A larger sample size would be desirable to substantiate the results of the present study, and longitudinal assessments are required to determine the stability and reliability of the results. An accurate method for evaluating hard tissue changes after periodontal therapy is still under investigation. The re-entry procedure appears to be the gold standard to date, while no single method can produce similar information consistently. The images provided by the CBCT technique, combined with clinical measurements, will certainly prove useful, thereby avoiding the re-entry procedure.

## Conclusion

It can be concluded, within the limits of the study, that the use of 1.2% ATV gel as an adjunct to SRP is more beneficial in achieving better results in terms of periodontal regeneration. Being a noninvasive procedure, it serves to be an attuned way to treat periodontal intraosseous defects, leading to a functionally more stable masticatory apparatus.

## Acknowledgement

The authors thank Dr. Puranik, the Head of the Department of Pharmacology, Rashtriya Sant Tukdoji Maharaj University, Nagpur, for the help in the procurement and preparation of the Atorvastatin gel.

## Authors’ Contributions

The authors contributed in the following way.PYS was responsible for the definition of the intellectual content, literature search, clinical studies, experimental studies and data acquisition and interpretation. APK was responsible for the concept and design of the study, literature search, data analysis and manuscript preparation as well as editing. RAK contributed to the design, definition of the intellectual content, literature search, clinical studies and final approval of the version to be submitted. PVB was responsible for drafting and critically revising the article for important intellectual content and manuscript editing.

## Funding

This research did not receive any specific grant from the funding agencies in the public, commercial, or not-for-profit sectors.

## Competing Interests

The authors declare no competing interests with regards to the authorship and/or publication of this article.

## Ethics Approval

Institutional Ethical Committee Reference Number: IEC/VSPMDCRC/15/2016.
